# Adoptive parents’ finances and employment status: a 5-year longitudinal study

**DOI:** 10.1007/s00787-022-01946-3

**Published:** 2022-01-22

**Authors:** Amy L. Paine, Kevin Fahey, Rebecca Thompson, Katherine H. Shelton

**Affiliations:** 1 School of Psychology, Cardiff University, Tower Building, 70 Park Place, Cardiff, CF10 3AT UK; 2 School of Politics and International Relations, University of Nottingham, University Park, Nottingham, NG7 2RD UK

**Keywords:** Adoption, Income, Employment, Mental health

## Abstract

We investigated risk and facilitating factors related to families’ change in finances and employment over 5 years following adoption of a child from local authority care in a prospective, longitudinal study of children placed for adoption between 2014 and 2015 (*N* = 96). Parents completed questionnaires at approximately 5, 21, 36, 48 and 60 months post-placement. We used time series analysis to examine the impact of child (e.g. pre-placement experiences, mental health), family structure (e.g. number of siblings, parent relationship status), and parent (e.g. mental health) factors on change in household income and parent employment status after adoption. We also examined the tendency for parents to comment on employment and finances and the emotional valence of their comments to gauge their concern about their circumstances. Children’s mental health problems were associated with primary caregivers reducing their time spent in employment and parents’ tendency to comment on their financial and work circumstances. Children who experienced more moves in care were more likely to have a primary caregiver not in full-time work, as were children with higher prosocial behaviour scores. Being in full-time work was associated with parents’ symptoms of anxiety. We also detected associations between structural features of the family and changes in income and employment. This study represents one of the first empirical investigations of factors associated with the socioeconomic features of adoptive families’ lives and informs ongoing discussion regarding the support needs of families and the timing, nature, and delivery of post-adoption professional services.

**Supplementary Information:**

The online version contains supplementary material available at 10.1007/s00787-022-01946-3.

## Introduction

The birth of a child represents an important transition for all families [1, 2], but for families who adopt, becoming a parent involves navigating numerous unique obstacles and challenges. Many parents choose to adopt following experiences of infertility and may navigate parenthood at an older age [3]. In the UK, adopting a child can be a lengthy and challenging process involving parental fitness evaluations, waiting to be matched and, potentially, difficulties encountered in the context of court processes and contested applications by birth parents [4, 5]. Following placement of their child, adoptive families face many of the challenges associated with new parenthood, but with an increased likelihood of parenting a child with emotional and behavioural difficulties [6, 7]. Adoptive parents may have to make significant adaptations to meet the emotional and behavioural needs of their child. This may include parents reducing their working hours and earnings to support and spend time with their child, as well as liaising with teachers, healthcare, and other professionals. [8]. Although evidence suggests that newly formed adoptive families might experience financial strain [8], to our knowledge, no studies appear to have examined the risk and facilitating factors that are related to change in employment and finances after adoption. To identify the support needs of and inform provision of services to families who adopt in the UK, we investigated child, parent, and family factors associated with change in parent employment and household income over 5 years following placement of a child from state care for adoption.

It is well established that how parents manage their finances, employment, and parental leave to care and provide resources for their child and family impacts their adaptation to parenthood [2]. Like approaches to understanding transitions to biological parenthood, models that consider how adoptive families adjust to the placement of a child emphasise interactional processes between the demands parents face (heightened risks and stressors) and their available resources (physical, psychological); and consider how families encounter difficulty when demands outweigh resources (e.g. [9]). Demographic profiles of adoptive families suggest that they tend to be more financially secure [3, 10]. However, families also report financial loss [11], and although adopters’ strong commitments to their children make it difficult for them to acknowledge a need for financial support, previous research has demonstrated that a third of adopters describe additional support (i.e. adoption allowance) as ‘vital’ [12]. Further, financial difficulties are often raised as a stressor for adoptive parents [11, 13] and are associated with a higher risk of the adoptive placement reaching crisis point [14]; including *disruption*; where a legally adopted child leaves the family prior to the age of 18 [11].

Although recent evidence suggests a lower prevalence rate of mental disorders for adopted children compared to those who experience other care settings (i.e. foster, kinship, or out-of-home care; [15]), children adopted from the UK public care experience higher rates of emotional and behavioural problems than population norms [16]. One identified source of strain for adoptive families is children presenting with challenging behaviour [11]. Indeed, children adopted from public care underperform across a range of domains of neurocognitive development [17, 18] and have a greater risk of developing mental health problems compared to their non-adopted counterparts [6, 7] that endure in the years following their adoptive placement [19]. In the UK, children are removed from the family home when it is determined their birth family is unable to care for them [11], and most spend time in temporary kinship or foster care [20]. Studies indicate that adoptees’ mental health problems are, at least in part, attributable to their pre-adoptive history, such as their early experiences of adversity (in most cases, abuse or neglect, [21, 22]) and instability in living arrangements [23], where children adopted later in childhood and in sibling groups are likely to experience more severe problems associated with spending more time with their birth family and in care [24–26]. Given adopted children’s early experiences and support needs [27, 28] and that, primarily, parents seek additional support with the aim of improving their child’s emotional health and wellbeing [29], it is likely that some parents decrease their work hours (to the possible detriment of their financial situation) to meet the psychological needs of their child(ren) [8, 24].

Parenting an adopted child with mental health problems is associated with parents’ own reports of distress [30]. Although many adopters may transition to parenthood with more psychological and physical resources available to them [31, 32], adoptive parents experience higher than general population levels of depression and anxiety symptoms [30, 33]. Adoptive parents’ concerns include changes in lifestyle, routines, and financial difficulties that are associated with concerns about the future [11, 13]. Additionally, adoptive parents’ financial problems place strain on their close relationships (i.e. with their spouse or partner, [34]) and are associated with reductions in self-care [35]. In addition to examining child-related factors in relation to changes in adoptive family circumstances—such their mental health and pre-adoptive history—we also investigated associations between adoptive parent mental health and changes in employment and income.

## The present study

This is the first prospective, longitudinal study to investigate the impact of child, parent, and family factors associated with the financial and work-related behaviours of parents of domestically adopted children in the UK. We followed families who adopted a child from local authority care five times over 5 years post-adoptive placement. First, we employed *time series* analytical methods to investigate both concurrent and lagged variables associated with changes in household income and employment status over 5 years. We hypothesised that children’s pre-adoptive adversity (indicated by number of adverse childhood experiences; [16, 36] and number of moves in care), being in a sibling group, and emotional and behavioural problems would be associated with a reduction in household income and parent tendency to be in full-time employment. We also hypothesised that we would detect negative associations between parents’ symptoms of anxiety and depression and their household income.

In addition to examining factors associated with change in family finances and employment over time, we wanted to capture the level of adoptive parents’ concern about their circumstances. Therefore, secondly, we used *sentiment analysis* to examine the emotional valence (positive or negative) of all comments made by adoptive parents regarding their employment and finances from open-ended responses across the five waves of assessment. We hypothesised that child pre-adoptive adversity, sibling status, and emotional and behavioural problems, and parents’ symptoms of anxiety and depression would be associated with comments with negative valence regarding finances and employment.

## Methods

### Design

The Wales Adoption Cohort Study used a prospective, longitudinal mixed-methods approach to understand the early support needs and experiences of 96 newly formed adoptive families. Local authority adoption teams across Wales were asked to send out letters on behalf of the research team to every family with whom they had placed a child for adoption from 01 July 2014 to 31 July 2015 (see [10] for more details of the study and [19, 37] for background of adoption in the UK). The 96 families who returned the initial questionnaire at 5 months post placement were followed up longitudinally over five time points post-placement. The present study focuses on the questionnaire follow ups that took place at approximately 5, 21, 36, 48 and 60 months post-placement (Waves 1 to 5 [W1 to 5] respectively). Of the 96 families that participated in the study at W1, 81 (84.4%) at W2, 73 (76.0%) at W3, 68 (70.8%) at W4, and 63 (65.6%) at Wave 5. This study was conducted in accordance with the 1964 Declaration of Helsinki and its later amendments. Ethical permission for the study was granted by the Research Ethics Committee for the School of Psychology at Cardiff University and permission to access the social work records was obtained from the Welsh Government (see [16] for more details). STROBE compliance for this study [38] was assessed and is presented in Supplementary Materials.

### Procedure

#### Child adoption reports (CARs)

Within Wales, every local authority is mandated to complete a Child Adoption Report (CAR), for each child where there is a plan for adoption, as set out in the Adoption Act Regulations (2005). CARs are completed by social workers, who record information based on their work with birth parents, contact with foster carers, liaison with other professionals (e.g. police, health visitors, and medical officers), and reviews of historical social services records. Baseline data concerning the child characteristics and the pre-adoptive history of each child were obtained by reviewing these records. Researchers worked on-site at the local authority offices and gathered information pertaining to the pre-adoptive history of the child and the age at which the child was moved into their permanent placement from electronic and hard-copy formats of CAR records.

#### Questionnaires

At each time point, families completed a questionnaire concerning sociodemographic information, pre- and post-adoption experiences, the child and parent’s mental health, and adoptive family relationships. Questionnaires pertaining to socio-demographic information were developed by the study team. Where groups of siblings were placed together, parents were asked to report on the eldest child in the placement. Questionnaires were completed by either an adoptive mother (87.5% at W1, 87.7% at W2, 97.3% at W3, 92.6% at W4, 90.5% at W5) or father. It was encouraged that the questionnaires should be completed by the same parent at each wave, so all families who provided follow-up questionnaires returned at least one completed by the same informant.

### Participants

Of the children who were reported on by their parents in the longitudinal follow-up questionnaires (*N* = 96), 47 (49%) were female, and were placed for adoption at a median age of 2 years (IQR = 1–3). Children spent a mean of 1.43 (SD = 1.68, range 0–6.42) years with their birth parents and a mean of 1.47 (SD = 0.78, range 0.56–3.84) years in care. Children experienced a median of 2 moves (IQR = 1 to 4) whilst in care. Twenty-nine children (30%) were adopted as part of a sibling group.

The adoptive parents in the study were a mean age of 40.67 (SD = 6.99, range 22–62) years at the time of adoption, and the majority (97.9%, *n* = 94) were white British. Most parents were in a heterosexual relationship (82%, *n* = 79), 5% (*n* = 5) were in a same sex relationship and 13% (*n* = 12) were single adopters. At the W1 assessment, there were a mean of 3.65 (SD = 1.02, range 2–7) people living in the household and most informants were in either full-time or part-time paid work (*n* = 72, 54.2%). Gross family income and education levels were substantially higher than the UK average (see [16]); 12% earned more than £75,000 per year and 37% had postgraduate degrees.

### Sample representativeness

Characteristics of the 96 adopted children in the present study were compared to those of all children placed for adoption in Wales the same time window (*N* = 374), by reviewing CARs for all children adopted between July 2014 and July 2015 in Wales. The sample was representative of children placed for adoption in this 13-month period regarding gender and past experiences of abuse and neglect (*p*s > 0.05). However, it contained slightly older children because we asked parents of sibling groups (30% of the sample) to comment on the oldest child they had adopted. Attrition analyses showed no differences in sociodemographic characteristics (child gender and age, parent age, relationship status, education, income, and ethnicity) between those who participated in W1 and 5 of the study (all *p*s > 0.05, see Online Supplement for socio-demographic characteristics of sample at W1 and W5 and for survival model outputs of main variables of interest).

### Measures

#### Adoptive parent income and employment

Parents provided information on their income and employment in the questionnaires. Parents indicated their household income before tax from options of (1) up to £10,000, (2) £10,000–£19,999, (3) £20,000–£29,999, (4) £30,000–£49,999, (5) £50,000–£74,999, or (6) £75,000 + . Parents were not asked about their income at Wave 2; therefore, we excluded Wave 2 from any analysis including income. At each wave, parents provided information on their own and if applicable, their partner’s employment status, where 0 = unemployed, 1 = part-time, 2 = full-time. For each adoptive parent, we coded change in employment on a 5-point Likert-scale (− 2 = change from full-time employment to unemployed, − 1 = change from part-time employment to unemployed/full-time to part-time; 0 = no change in employment, 1 = change from unemployment to part-time work/part-time to full-time, and 2 = change from unemployment to full-time work).

#### Adoptive parent open-ended responses regarding income and employment

All comments regarding finances and employment were extracted from open-ended responses across all questionnaires at each wave of assessment. We used two measures: (1) the count of open-ended responses at each wave; and (2) an aggregated score of the emotional valence of comments. Each comment regarding finances and/or employment were assessed using *sentiment analysis* [39] which uses a pre-defined dictionary to identify positive and negative words (we used the sentiments function in the tidytext package in R). Comments were coded as − 1 = negative, 0 = neutral, and 1 = positive, and an aggregate score was computed for each participant across all their comments made at each wave.

#### Adoptive parent characteristics and family structure

Additional adoptive parent sociodemographic information was collected at W1. Variables included: (1) adoptive parent (questionnaire informant) age at time of adoption; (2) adoptive parent relationship status (1 = single adopter, 2 = couple adopter); (3) adoptive parent highest level of education attained (1 = postgraduate or higher degree, 0 = other); (4) whether children were adopted alone into the household or whether other children were in the household (1 = any sibling, 0 = no sibling).

#### Pre-adoptive risk factors

Information regarding child characteristics (gender and date of birth) and their pre-adoptive background were obtained from review of each child’s CAR. Pre-adoptive risk factors included: (1) child’s age at placement in years; (2) number of moves, defined as any change in living arrangement deemed significant by the child’s social worker prior to their adoptive placement; and (3) number of adverse life experiences (ACEs) out of ten categories, see [32, 33], including childhood abuse (emotional, physical, or sexual), neglect, and household dysfunction (domestic violence, parental separation, substance abuse, alcohol abuse, mental illness, or incarceration). Each category was coded as absent (0) or present (1) resulting in an aggregate ACEs score for each child of 0–10.

#### Child emotional and behavioural problems and prosocial behaviour

Adoptive parents completed the Strengths and Difficulties Questionnaire [40] at each wave of the study. We used emotional (internalising) problems (sum of emotional and peer problem scales), behavioural (externalising) problems (sum of conduct and hyperactivity scales), and prosocial behaviour as our key outcome variables. A higher score is indicative of more problems for all subscales, except for the prosocial scale, where higher scores correspond to strengths in prosocial behaviour (where children could score a maximum of 20 for emotional and behavioural problems, and 10 for prosocial). The emotional, behavioural, and prosocial scales had acceptable to good levels of internal consistency across all time points (*α*s ranged from 0.60 to 0.84).

#### Parent symptoms of depression and anxiety

The Hospital Anxiety and Depression Scale (HADS) is a brief 14-item self-report measure of anxiety and depression [41] and was completed by adoptive parents at each wave of the study. The scale comprises 14 items, seven of which assess anxiety (e.g. “I can sit at ease and feel relaxed”) and seven which measure depression (e.g. “I still enjoy the things I used to enjoy”). The items are scored on a 4-point scale ranging from 0 to 3, with higher scores indicating higher anxiety and depression (maximum score is 21 on each scale). The HADS has good discriminant validity, internal consistency, and concurrent validity [42]. The HADS was completed at all five time points and had good levels of internal consistency (depression; *α* = 0.741–0.798 and anxiety; *α* = 0.808–0.860).

### Statistical analysis

The combined dataset consisted of 381 observations across five waves with a mean inter-wave attrition rate of 10.16 percentage points. The unit of observation was the respondent at each wave. Where families were couple adopters, we designated a ‘Parent 1’ (most often the informant of the questionnaire) and a ‘Parent 2’. Our preliminary analyses showed that there was very little tendency for caregivers designated as ‘Parent 2’ to change their employment status over 5 years following the adoptive placement (see Supplementary Information) and that the reduction of ‘Parent 2 s’ being in full-time employment was predominantly related to families dropping out of the study over time. Therefore, our analyses regarding adoptive parent employment status focused on ‘Parent 1’ (hereafter referred to as the ‘Primary Caregiver’). We used three outcome variables in this study to assess factors associated with changes in income and employment status. Second, we investigated the text of open-ended responses regarding employment and income, to examine factors associated with parents’ propensity to comment on their finances and employment and their work status and the emotional valence of their comments.

We reported estimated coefficients for income and employment measures separately. Our estimation technique was ordered logistic regression and logistic regression maximum-likelihood estimation techniques, to account for the nature of the income and employment variables. When our outcome measures are derived from open-ended text responses, we use negative-binomial maximum-likelihood estimation (as our measure of the count of responses is over-dispersed), and ordinary least squares regression.

#### Modelling time

Our main research questions were addressed using R 4.1.2 [43], using conventional significance levels (*p* < 0.05). Due to the small sample size, we also reported findings where *p* < 0.10. Our estimation technique was ordinary least squares regression, accounting for serial correlation by employing time series analysis [44, 45]. Serial correlation violates the assumption that observations are independent; errors associated with an individual at time_t_ are positively correlated with errors at time_*t*-1_. We employed autoregressive distributive partial-adjustment lag model (AR-1) to overcome the problems with serial correlation. AR-1 models control for time by including the individual’s outcome from the previous time period as a predictor. This approach controls for different ‘starting points’ for each individual child and permitted us to estimate the outcome without concerns pertaining to each child having a different ‘starting point’. Thus, estimated coefficients observed effects of predictors on the outcome after controlling for the development of the child as a function of time. This analysis permitted comparison between long-run effects (the pre-adoptive experiences of adopted children in the study) and short-run effects (changes in the employment status or household income of parents or the unobserved contemporaneous wellbeing of the child). We were unable to conclusively test for co-integration due to sample size limitations and the relatively small number of waves in the study; therefore, we did not use error-correction models. Moreover, because many meaningful covariates were time invariant, we did not advocate for or use a differenced model.

#### Multiple imputation

We used the Amelia II R package to impute missing data [46]. We restricted imputed data to positive integers or zero. We also imputed our outcome variables when the respondent completed the questionnaire but did not fill out all questions related to our outcome variables; see the online supplement for robustness checks that do not impute missing data. However, as we cannot impute an entire wave of questionnaires, we exclude Wave 2 when our outcome variable is income, and the lag of income for Wave 3 is Wave 1.

## Results

Families’ household income reported at each time point in the study is presented in Table 1. At 5 months post-placement (W1), 56 (60.9%) primary caregivers were in full-time work, 24 (30.8%) were in full-time work at 21 months post-placement (W2), 27 (38.6%) at 36 months (W3), 21 (30.9%) at 48 months (W4), and 24 (38.1%) at 60 months (W5). Table 2 describes the tendency for primary caregivers to change their employment arrangements over time. Descriptive statistics for all variables of interest in the present study and correlations between all variables of interest are presented in Table 3.

**Table 1:** Frequencies and percentages of adoptive families’ household income brackets over 5 years following the adoptive placement

	Up to £10,000	£10,000 to £19,999	£20,000 to £29,999	£30,000 to £49,999	£50,000 to £74,999	£75,000 +	*N*
Approximate time following placement of child							
5 months (W1)	4 (4.2)	7 (7.3%)	14 (14.6%)	28 (29.2%)	31 (32.3%)	12 (12.5%)	96 (100%)
36 months (W3)	3 (4.1%)	3 (4.1%)	11 (15.1%)	25 (34.3%)	21 (28.8%)	10 (13.7%)	73 (100%)
48 months (W4)	3 (4.4%)	7 (10.3%)	4 (5.9%)	24 (35.3%)	18 (26.5%)	12 (17.6%)	68 (100%)
60 months (W5)	2 (3.2%)	4 (6.3%)	6 (9.5%)	20 (31.7%)	18 (28.6%)	13 (20.6%)	63 (100%)

Information on household income was not collected at the 21-month (Wave 2) assessment

**Table 2:** Frequencies and percentages of primary caregivers’ changes in employment over 5 years following the adoptive placement

	− 2	− 1	0	1	2	Valid *N*
Approximate time following placement of child						
21 months (W2)	4 (5.2%)	21 (27.3%)	49 (63.6%)	3 (3.9%)	0 (0.0%)	77 (100%)
36 months (W3)	2 (2.9%)	4 (5.8%)	48 (69.6%)	12 (17.4%)	3 (4.3%)	69 (100%)
48 months (W4)	2 (3.0%)	10 (14.9%)	42 (62.7%)	12 (17.9%)	1 (1.5%)	67 (100%)
60 months (W5)	1 (1.6%)	7 (11.1%)	47 (74.6%)	7 (11.1%)	1 (1.6%)	63 (100%)

For each adoptive parent, we coded change in employment on a scale of − 2 to 2, where − 2 indicated a change from full-time employment to unemployed, −1 a change from part-time employment to unemployed/full-time to part-time, 0 no change in employment, 1 a change from unemployment to part-time work/part-time to full-time, and 2 a change from unemployment to full-time work. Employment details were not completed for *n* = 4 families at W2 and W3, and *n* = 1 family at W4

**Table 3:** Associations between variables of interest

	1	2	3	4	5	6	7	8	9	10	11	12	13	14	15	16	17
1. Household income	–																
2. Change in primary caregiver employment	0.08	–															
3. Caregiver full-time work	0.33*	0.40*	–														
4. Child emotional problems	− 0.05	0.03	0.01	–													
5. Child behavioural problems	0.12	− 0.08	0.03	0.52*	–												
6. Child prosocial behaviour	− 0.03	0.06	− 0.12	− 0.24*	− 0.33*	–											
7. Number of moves	− 0.03	0.02	− 0.14	− 0.02	− 0.10	0.12	–										
8. ACE count	− 0.01	0.04	0.03	0.23*	0.21*	0.13	0.43*	–									
9. Child age^	− 0.12*	0.01	− 0.01	0.26*	0.25*	0.12	− 0.08	0.64*	–								
10. Parent depression	− 0.01	0.02	− 0.01	0.34*	0.29*	− 0.16*	− 0.09*	0.19*	0.22*	–							
11. Parent anxiety	− 0.05	0.04	0.01	0.31*	0.31*	− 0.12*	− 0.12*	0.15	0.14	0.67*	–						
12. Child gender	− 0.10	0.03	− 0.07	− 0.14*	− 0.10	0.25*	− 0.10	− 0.02	0.04	− 0.05	− 0.03	–					
13. Child sibling status	0.12*	− 0.01	− 0.04	0.22*	0.12	− 0.12	0.23*	0.14*	0.08	0.06	0.00	− 0.10*	–				
14. Parent age^	− 0.10	0.04	− 0.11	0.15	0.17	− 0.06*	− 0.22*	0.11	0.31*	0.07	0.07	− 0.15*	− 0.08	–			
15. Parent relationship status	0.46*	− 0.02	0.16*	− 0.20*	− 0.08	0.17*	0.06	− 0.06	− 0.21*	0.02	0.03	0.10	0.10	− 0.32*	–		
16. Parent education	0.06	0.04	− 0.01	0.10	− 0.04	0.03	− 0.21*	− 0.13*	− 0.06	− 0.04	− 0.04	− 0.12	0.10*	0.20*	− 0.06	–	
17. Primary caregiver employment	0.37*	0.45	0.88	0.08	− 0.04	− 0.02	− 0.07	0.01	0.03	− 0.07	− 0.07	− 0.10	− 0.11	− 0.14	0.17*	− 0.03	–
Mean	4.23	− 0.06	0.41	4.47	7.63	7.24	2.09	2.72	2.82	4.83	6.48	0.54	0.55	40.99	0.87	3.81	1.24
SD	1.28	0.69	0.49	3.44	3.92	2.17	2.01	2.79	2.06	3.36	3.79	0.5	0.5	6.83	0.34	1.27	0.72

The correlation coefficients were generated using an imputed dataset. Child emotional and behavioural problems and prosocial behaviours, and parent symptoms of depression and anxiety are pooled across all five waves of the study

*p < 0.05

^^^At Wave 1

### Child, parent, and family factors associated with household income over time

The first model estimating coefficients associated with changes in household income is presented in Table 4, Model 1. Being in a relationship (*B* = 0.983, *p* < 0.05), and the primary caregiver being in employment (*B* = 0.788, *p* < 0.01) was associated with increases in household income over 5 years post-adoption. More highly educated parents were more likely to experience decreases in household income over time (*B* = 0.214, *p* < 0.05). We found that presence of a sibling was associated with increased household income over time (*B* = 0.672, *p* < 0.05).

**Table 4:** Estimated coefficients for associations between child, parent, and family variables and the household income employment status of primary caregivers

	Household income	Primary caregiver change in employment	Primary caregiver in full-time work
Child emotional problems	0.060 (0.058)	0.082 (0.053)	0.055 (0.063)
Child behavioural problems	− 0.005 (0.048)	− 0.099* (0.049)	− 0.061 (0.061)
Child prosocial behaviour	− 0.067 (0.110)	− 0.024 (0.090)	− 0.235* (0.099)
Child emotional problems_*t-1*_	− 0.017 (0.068)	− 0.126* (0.060)	− 0.050 (0.072)
Child behavioural problems_*t-1*_	0.078 (0.057)	0.057 (0.057)	− 0.009 (0.071)
Child prosocial behaviour_*t-1*_	0.142 (0.098)	0.069 (0.071)	0.106 (0.084)
ACE count	− 0.066 (0.073)	0.056 (0.066)	0.067 (0.075)
Number of moves	− 0.055 (0.080)	− 0.016 (0.050)	− 0.171^+^ (0.089)
Child age	− 0.138 (0.091)	− 0.059 (0.066)	0.069 (0.089)
Child gender	0.103 (0.343)	− 0.148 (0.208)	− 0.317 (0.316)
Sibling status	0.672* (0.290)	0.126 (0.221)	− 0.198 (0.329)
Parent depression	− 0.052 (0.058)	0.028 (0.046)	− 0.098 (0.064)
Parent anxiety	− 0.014 (0.049)	0.029 (0.042)	0.094^+^ (0.055)
Parent age	0.032 (0.024)	0.016 (0.014)	− 0.027 (0.030)
Parent relationship status	0.983* (0.491)	− 0.069 (0.317)	1.200^+^ (0.676)
Parent education	− 0.214* (0.106)	0.083 (0.082)	0.0001 (0.134)
Primary caregiver employment	0.788** (0.245)		
Household income	2.210** (0.298)		
Primary caregiver in full-time work_*t-1*_			1.980** (0.383)
Intercept			0.102 (1.720)
Obs	212	285	285
Log-likelihood	− 189.0	− 269.0	− 139.0
Akaike information criterion	423.69	577.70	313.10

AR-1 = autoregressive models. Coefficients are unstandardised to allow for direct interpretability. Standard errors are clustered by individual respondent and presented in brackets below the coefficients

^+^*p* < .10, *p < .05, **p < .01

### Child, parent, and family factors associated with changes in employment over time

We also estimated coefficients for variables associated with change in employment status over time (see Table 4, Model 2). We found that children’s concurrent behavioural problems were associated with parents’ reduced employment over time, where a one-unit increase in concurrent behavioural problems was associated with a 0.906 increase in the odds of reducing employment by one level (i.e. full-time to part-time/part-time to unemployed), *p* < 0.05. Additionally, lagged emotional problems were associated with reductions in parent employment, where a one-unit increase in emotional problems was associated with a 0.881 increase in the odds of reducing work status by one level by the next wave of assessment, *p* < 0.05.

### Child, parent, and family factors associated with parent being in full-time work

In Table 4, Model 3, we present estimated coefficients for variables associated with parents’ tendency to stay in full-time work. We found that children’s concurrent reported prosocial behaviours were associated with parents’ tendency to remain in full-time employment; children with higher levels of prosocial behaviour were less likely to have parents in full-time employment, where a one-unit increase in child concurrent prosocial behaviour was associated with a 0.790 decrease in the odds of their parent having full-time employment status, *p* < 0.05. Additionally, children who experienced more moves between households while in care were more likely to have parents who did not remain in full-time employment, where each additional move was associated with a 0.843 decrease in the probability of their parent remaining in full-time employment, *p* < 0.10.

Parents who were in a relationship at the beginning of the study were more likely to remain in full-time employment over time, where being in a relationship was associated with a 1.200 increase in the probability of staying in full-time employment, *p* < 0.10. Parent concurrent levels of anxiety was also associated with being in full-time employment: a one-unit increase in anxiety was associated with a 0.094 decrease in the probability of remaining in full-time employment, *p* < 0.10.

### Child, parent, and family factors associated with comments about finances and employment

We employed a series of parsimonious models to test our hypotheses; we did so for three important reasons. First, overfitting a model—particularly with a small sample size—carries significant threats to inference. Second, due to the small sample size, complex maximum-likelihood models with many covariates were often unable to identify a single global maximum and therefore did not converge. Third, even with imputation, some covariates we would have wanted to include, but omitted, lacked sufficient variation across some waves and contributed to concerns over separation.

In open-ended responses, parents made *M* = 0.895 (SD = 1.163, range 0 to 9) comments about their finances and employment situation, and the sentiment of their responses was *M* = 0.089, (SD = 0.089, range − 3 to 6). In Table 5, Model 1, we present the estimated coefficients for variables associated with count of comments regarding employment and finances. Lagged child emotional problems was associated with parents’ number of comments about employment and finances, where an increase of one level in child emotional problems was associated with a 1.028 increase in the odds of making a comment at the next wave of the study, *p* < 0.05.

**Table 5:** Estimated coefficients for associations between child, parent, and family variables and the number open-ended responses regarding income and finances and the valence of the open-ended responses

	Total open-ended responses	Emotional valence of open-ended responses
Child emotional problems	0.027 (0.028)	0.001 (0.035)
Child behavioural problems	0.020 (0.025)	0.009 (0.037)
Child prosocial behaviour	0.030 (0.038)	0.006 (0.060)
Child emotional problems_*t-1*_	0.070** (0.036)	− 0.005 (0.034)
Child behavioural problems_*t-1*_	− 0.003 (0.028)	− 0.062^+^ (0.034)
Child prosocial behaviour_*t-1*_	0.045 (0.047)	0.022 (0.073)
ACE count	− 0.033 (0.036)	0.036 (0.045)
Number of moves	− 0.033 (0.036)	− 0.048 (0.036)
Count of responses_*t-1*_	0.192* (0.077)	
Constant	− 1.440** (0.450)	0.212 (0.480)
Obs	285	150
Adjusted *R*^2^		0.001
Log likelihood	− 360.0	
Akaike information criterion	729.90	
*F* statistic		1.01

AR-1 = autoregressive models. Coefficients are unstandardised to allow for direct interpretability. Standard errors are clustered by individual respondent and presented in brackets below the coefficients. The lag of the number of questions is included to determine whether some respondents were more likely to provide open-ended responses. In the first model, individuals who did not provide a comment were given a value of 0 open-ended responses. In the second model, individuals who did not provide comments were removed from analysis

^+^*p* < .10, *p < .05, **p < .01

In Model 2, we show factors associated with the sentiment of parents’ comments about employment and finances. We found that lagged child behavioural problems were associated with the valence of parents’ responses, where a one level increase in child behavioural problems was associated with a 0.939 increase in the odds of the comments about finances and employment being negative at the next wave of the study, *p* < 0.10.

## Discussion

In the extant adoption literature, there has been an entirely appropriate focus on family relationship quality and the mental health of children and their parents after adoption (e.g. [10, 47]). This focus has revealed the highs and the challenges that accompany children joining a family from care. Although financial difficulties are raised by families as a source of stress post-adoption [11, 13], to our knowledge, no studies have examined factors associated with the socioeconomic features of families’ lives. We examined change, and factors associated with change, in adoptive families’ household income and employment arrangements over 5 years following the placement of a child from state care for adoption. We identified specific child and family factors that were associated with adoptive parents’ tangible changes in their household income and employment and of their tendency to comment on their family circumstances.

Many children adopted from public care experience enduring mental health problems [19, 25] or present with specific neurocognitive problems that may result in challenges in later childhood [17, 18]. We found that children’s emotional and behavioural problems were associated with primary caregivers’ tendency to change their employment arrangements over time; child behavioural problems were concurrently associated with primary caregivers reducing their time spent in employment, and child emotional problems were associated with primary caregivers changing their employment arrangements by the next time point of assessment. Children’s emotional problems were associated with an increased tendency for parents to comment on their finances and employment. Where children presented with more behavioural problems, parents made more negative comments about finances and employment. Our findings indicate that not only are tangible changes being made in relation to adopted children’s mental health needs, but that parents are preoccupied by, or else cognizant of this; for example, one parent commented: “Nursery concerned and close to excluding [child] due to daily events… I have been looking for alternative job with less hours.” Our analyses highlight the vital importance of families’ access to both psychological *and* practical resources, not only initially after placement, but as needs emerge. One family commented on the value of this, “Adoption allowance and funded play therapy has enabled me to afford not to work and focus on meeting my [child’s] needs.” Our findings also indicate that parenting interventions that have positive effects on child behaviour problems (for a meta-analytic review, see [48] have the potential to be a preventative strategy for adoptive parents’ financial problems and concerns about employment. More research is needed to examine this possibility.

Children who experienced more moves while in care were more likely to have a primary caregiver who opted not to remain in full-time work. Given that children who experience greater instability in their living arrangements following removal from their birth family are at greater risk for developing mental health problems [49], this might suggest a tendency for primary caregivers to seek to meet their child’s need for stability. As this was a concurrent association, we cannot determine causality or directionality from this finding, although it suggests that some children may experience some remedial benefit from having their primary caregiver at home with them for longer periods of time. Yet to better understand processes by which time with primary caregivers may foster better outcomes for children, more emphasis in future research should be placed on the *quality* of parent child interactions; particularly given that warm parenting that is sensitive, nurturing, and responsive, is associated with a marked decrease in adopted children’s mental health problems [10]. Future research should investigate the mechanisms by which parents’ and children’s time spent together may be associated with better outcomes for adopted children.

Parents’ symptoms of anxiety were positively associated with their being in full-time work. This study, however, does not capture the mechanisms that might explain this relationship. It is possible that this association results from strain in having to juggle family life and work obligations, perhaps resulting in less time for respite and self-care [35] and strain on their close relationships (i.e. with their spouse or partner; [34]). Families reflected upon these reasons in their comments, “It would be better if there was financial support so I could work part time. [The child] would benefit from more time with us, not after school etc. Also, I’d get more time to recoup and stay fit.” Given that adoptive parents experience higher than population levels of anxiety and depression that remain relatively stable across time [30], in addition to ensuring families are aware of available practical support and pathways for seeking support for their child’s mental health needs, their own mental health must be supported.

We detected associations between family structure and financial and work circumstances. Contrary to our expectations, having siblings was associated with greater household income over time. This may reflect that sibling placements involve older children of school age and parents are therefore able to return to employment sooner. It may also reflect a selection effect, where families with multiple children may have greater initial resources regarding income or added financial security. Unsurprisingly but importantly, being in a relationship was associated with the primary caregiver being less likely to be in full-time work and higher household income over time, suggesting that dual income families may be more able to facilitate one parent spending less time in employment. Although many families comprised of single parent adopters thrive [50], some single parents may have less opportunity or available resources to be flexible in their employment arrangements compared to dual parent families. These findings highlight the importance of long-term practical support for single adopters, as underscored by their comments, “I still believe that there should be financial support offered to all adopters, but especially to single adopters to take the pressure off the need to work. The first year would be essential… but even 2–3 years on the child needs to be the priority and I should not struggle financially in order to focus on my child.” Adopters also reflected on the time constraints and psychological strain associated with single parenthood, for example, one commented: “I can see many advantages to being a single parent but have recently realised that I’m having very very limited ‘me’ time. I’m starting to feel exhausted.” These findings emphasise the importance of ensuring single adopters receive information and are aware of pathways for seeking practical and psychological support for themselves and their children.

## Strengths and limitations

The covariates included in the present study enabled us to begin to disentangle the impact of various factors on adoptive family life and home economics over five annual occasions. However, a more fine-grained assessment of salaries, finances, allowances, and management of parental leave would provide further insight into how adoptive families’ financial circumstances and employment arrangements change. Our investigations were also conducted in the context of a relatively small sample, and future investigations should be conducted in the context of a larger samples to enable investigations of bidirectional processes by which these factors may be linked; for example, in addition to child mental health being associated with changes in circumstances, family circumstances such as financial strain may also affect child mental health via its impact on parent mental health [51]. Additionally, our study did not take full account of the informal but likely invaluable support provided by family, friends, and the local community [52]; indeed, families commented on the role of their support networks, “Emotional support, advice, sharing childcare, help with laundry, financial support—I have a strong network of family and friends who help out on a daily basis in one way or another.” Therefore, future research regarding adoptive families’ changing circumstances should take their sources of emotional and practical support into consideration.

## Conclusion

Our findings reveal factors that appear to have meaningful implications for the interface between work and family life, including employment, income, and contractual arrangements. We found evidence of links between children’s profile of mental health and parents’ employment, and overall, there was a sense of the capacity for experiences at home to spill over into comments about employment arrangements and financial security. Underlying this relationship is likely to be parents’ overriding desire to achieve positive outcomes for their children in the long term, together with the time and effort required to advocate for professional support to meet their child’s needs in the short term. Our findings underscore the pervading effects of children’s mental health on family life. Adoptive parents need support to consider and anticipate the potential conflict between the competing needs of employers and children, with particular emphasis on ensuring that their own psychological health is maintained.

## Supplementary Information

Below is the link to the electronic supplementary material.

Supplementary file1 (DOCX 87 kb)
